# Salt Stress Mitigation via the Foliar Application of Chitosan-Functionalized Selenium and Anatase Titanium Dioxide Nanoparticles in Stevia (*Stevia rebaudiana* Bertoni)

**DOI:** 10.3390/molecules26134090

**Published:** 2021-07-05

**Authors:** Morteza Sheikhalipour, Behrooz Esmaielpour, Gholamreza Gohari, Maryam Haghighi, Hessam Jafari, Habib Farhadi, Muhittin Kulak, Andrzej Kalisz

**Affiliations:** 1 Department of Horticulture, Faculty of Horticulture, University of Mohagheh Ardabili, Ardabil 13131-56199, Iran; peyman.sh.alipour@gmail.com (M.S.); behsmaiel@yahoo.com (B.E.); 2 Department of Horticulture, Faculty of Horticulture, University of Maragheh, Maragheh 55181-83111, Iran; h.farhadi.14@gmail.com; 3 Department of Horticulture, College of Agriculture, Isfahan University of Technology, Isfahan 84156-83111, Iran; mhaghighi@cc.iut.ac.ir; 4 Department of Organic Chemistry, Faculty of Chemistry, University of Tabriz, Tabriz 51666-16471, Iran; hessamjafari1996@gmail.com; 5 Department of Herbal and Animal Production, Vocational School of Technical Sciences, Igdir University, Igdir 76000, Turkey; muhyttynx@gmail.com; 6 Department of Horticulture, Faculty of Biotechnology and Horticulture, University of Agriculture in Krakow, 31-120 Krakow, Poland

**Keywords:** abiotic stress, antioxidant enzymes, essential oil, nanotechnology, oxidative stress, steviol glycosides

## Abstract

High salt levels are one of the significant and major limiting factors on crop yield and productivity. Out of the available attempts made against high salt levels, engineered nanoparticles (NPs) have been widely employed and considered as effective strategies in this regard. Of these NPs, titanium dioxide nanoparticles (TiO_2_ NPs) and selenium functionalized using chitosan nanoparticles (Cs–Se NPs) were applied for a quite number of plants, but their potential roles for alleviating the adverse effects of salinity on stevia remains unclear. Stevia (*Stevia rebaudiana* Bertoni) is one of the reputed medicinal plants due to their diterpenoid steviol glycosides (stevioside and rebaudioside A). For this reason, the current study was designed to investigate the potential of TiO_2_ NPs (0, 100 and 200 mg L^−1^) and Cs–Se NPs (0, 10 and 20 mg L^−1^) to alleviate salt stress (0, 50 and 100 mM NaCl) in stevia. The findings of the study revealed that salinity decreased the growth and photosynthetic traits but resulted in substantial cell damage through increasing H_2_O_2_ and MDA content, as well as electrolyte leakage (EL). However, the application of TiO_2_ NPs (100 mg L^−1^) and Cs–Se NPs (20 mg L^−1^) increased the growth, photosynthetic performance and activity of antioxidant enzymes, and decreased the contents of H_2_O_2_, MDA and EL under the saline conditions. In addition to the enhanced growth and physiological performance of the plant, the essential oil content was also increased with the treatments of TiO_2_ (100 mg L^−1^) and Cs–Se NPs (20 mg L^−1^). In addition, the tested NPs treatments increased the concentration of stevioside (in the non-saline condition and under salinity stress) and rebaudioside A (under the salinity conditions) in stevia plants. Overall, the current findings suggest that especially 100 mg L^−1^ TiO_2_ NPs and 20 mg L^−1^ Cs–Se could be considered as promising agents in combating high levels of salinity in the case of stevia.

## 1. Introduction

Salt stress is one of the widely investigated abiotic stress factors because of its clearly reported adverse effects on plant growth and development, as manifested in crop yield loss [1,2,3,4]. Regarding osmotic adjustment, ion toxicity and oxidative stress, the negative effects of salt stress were reported for a quite number of plant species in recent years [5,6,7,8]. These reports suggest that most of the agronomically important crop species are not compatible with high salt levels, as uttered in a review by Parihar et al. [4]. Furthermore, the Food and Agriculture Organization (FAO) reports stated that salinity is a serious threat to over 6% of the world’s land [4] and the relevant reports anticipate that salinity will severely affect more than 50% of arable land by 2050 [9]. For this reason, the use of new methods and strategies to decrease the negative effects of salinity has gained great importance. Of the recent attempts undertaken, nanoscale materials were employed as a nanotechnological approach and accordingly exhibited protective roles for quite a number of crop plants, suggesting the better tolerance of plants against the harsh conditions [10,11].

Nanotechnology is the study and application of nanoscale particles with specific qualities and small diameters (1–100 nm); they are widely applied in various agriculture aspects, such as plant nutrition, plant protection and nanopesticides [12]. The relevant studies regarding functionalization, modification or newly conjugated structures of nanoparticles are some of the emerging and hot topics nowadays [10]. Out of the available nanomaterials, cerium dioxide (nCeO_2_) [13,14,15,16], magnetite (nFe_3_O_4_) [17,18,19], zinc oxide (nZnO) [20], silicon dioxide (nSiO_2_) [21], copper oxide (nCuO) [22], aluminum oxide (nAl_2_O_3_) [23] and carbon nanotubes [24] showed protective roles in plants under stress conditions. In the case of medicinal and aromatic plant species, the application of nanoparticles is one of the novel and wise strategies used to increase growth, yield and especially secondary metabolites in plants under salt stress [25]. Moreover, selenium functionalized by a chitosan nanocomposite (Cs–Se NPs) and titanium dioxide nanoparticles (TiO_2_ NPs) are some of the proven substantial molecules that are used to enhance the abiotic tolerance range of the crop via the activation of defense mechanisms [26,27]. Considering the relevant stress indicators, treatments of TiO_2_ NPs and Cs–Se NPs decreased MDA and H_2_O_2_ content and increased agronomic parameters, photosynthetic pigments content, chlorophyll fluorescence, soluble sugars, proline content and antioxidant enzymes activity in some plant species under salinity stress [25,27,28]. In addition to the enhanced crop productivity under salinity stress, total phenolic compounds and essential oil yield were also increased with TiO_2_ NPs and Cs–Se NPs treatments [25,27,29]. However, high TiO_2_ NP concentration decreased the growth parameters, photosynthetic pigments and antioxidant activity and increased MDA and H_2_O_2_ in the plant [25,28].

Stevia (*Stevia rebaudiana* Bertoni) belongs to the Asteraceae family and is a reputed medicinal plant, possessing diterpenoid steviol glycosides (i.a., stevioside and rebaudioside A), which are 300 times sweeter than sucrose. For this reason, stevia is known as sweet leaf or sugar leaf. Since the human body does not metabolize the glycosides of stevia, stevia contains zero calories. This property of stevia leads the plant to be assessed as a natural sweetener to control diabetes. In order to reveal the changes of these metabolites and other relevant biochemical responses of stevia against salt stress, a wide array of well-documented studies were undertaken [30,31,32,33,34,35]. However, to our best knowledge, no studies have hitherto investigated the effects of TiO_2_ NPs and Cs–Se NPs on stevia grown under salt stress. Corresponding to the enhancing and stimulating roles of elements and their application as nanoparticles at non-toxic concentrations, we hypothesized that the relevant nanoparticles will improve the antioxidant status, steviol glycosides and other physiological parameters of stevia; furthermore, these improvements will be manifested in the growth and development of stevia. Both nanoparticle types were previously tested [25,27] and produced promising results related to plant growth, the alleviation of salt stress and an increase in plant chemical constituents desired by humans. As deduced from the findings from the studies by Gohari et al. [25] and Sheikhalipour et al. [27], three concentrations of TiO_2_ NPs (0, 100 and 200 mg L^−1^) and Cs–Se NPs (0, 10 and 20 mg L^−1^) were assayed for the current study. Therefore, our research group set out to test the effects of these molecules on a new valuable plant species, namely, stevia, as part of a series of studies in this research area.

## 2. Results

### 2.1. Effect of Cs–Se NPs and TiO_2_ NPs on Plant Growth Parameters

Salinity significantly decreased the plant growth parameters, where higher salt levels decreased the shoot height by 40.83%, root height by 37.45%, shoot fresh weight by 62.67%, root fresh weight by 51.26%, shoot dry weight by 64.11% and root dry weight by 50.68% as compared with the non-saline condition (Table 1). However, the application of TiO_2_ NPs and Cs–Se NPs significantly increased the plant growth parameters under the saline conditions. The current findings revealed that the Cs–Se NPs (20 mg L^−1^) treatment positively affected the shoot height (17.80% and 18.94%), root height (9.82% and 13.32%), shoot fresh weight (16.26% and 16.43%), root fresh weight (7.16% and 11.52%), shoot dry weight (17.87% and 23.70%) and root dry weight (7.07% and 13.47%) under the 50 mM and 100 mM salt levels, respectively, in comparison with the non-NP-treated plants.

Out of the applied TiO_2_ NP levels, TiO_2_ NPs (100 mg L^−1^) significantly improved the shoot height (14.94% and 17.27%), root height (8.92% and 11.30%), shoot fresh weight (11.19% and 16.74%), root fresh weight (5.92% and 10.66%), shoot dry weight (12.74% and 23.94%) and root dry weight (6.17% and 11.07%) under the 50 mM and 100 mM salt levels, respectively, in comparison with the non-NP-treated plants (Table 1).

### 2.2. Effect of Cs–Se NPs and TiO_2_ NPs on Photosynthetic Pigments, Net Photosynthetic Rate (Pn) and Maximum Quantum Efficiency of Photosystem II (Fv/Fm)

Salinity significantly decreased chlorophyll *a* and *b*, total chlorophyll, carotenoid concentrations, Pn and Fv/Fm (Table 1). However, the TiO_2_ NPs and Cs–Se NPs exhibited positive effects on the photosynthetic pigments, Pn and Fv/Fm under salinity. The current findings showed that a 100 mM salinity concentration decreased chlorophyll *a* by 46.63%, chlorophyll *b* by 23.46%, total chlorophyll by 43.96%, carotenoid by 21.99%, Pn by 22.42% and Fv/Fm by 15.48% in comparison with the non-saline condition. Cs–Se NPs (20 mg L^−1^) increased content of chlorophyll *a* (12.18% and 15.38%), chlorophyll *b* (2.73% and 7.70%), total chlorophyll (11.07% and 14.04%), carotenoid (6.72% and 8.71%), Pn (5.82% and 7.96%) and Fv/Fm (3.38% and 10.27%) in comparison with non-treated plants under the 50 mM and 100 mM salt levels, respectively. Furthermore, TiO_2_ NPs (100 mg L^−1^) significantly enhanced the concentration of chlorophyll *a* (11.04% and 10.73%), chlorophyll *b* (3.92% and 8.11%), total chlorophyll (10.21% and 10.22%), carotenoid (7.05% and 7.05%), Pn (4.21% and 6.64%) and Fv/Fm (1.46% and 8.64%) parameters in comparison with non-treated plants under the 50 mM and 100 mM salt levels, respectively (Table 1).

### 2.3. Effect of Cs–Se NPs and TiO_2_ NPs on Proline Content and RWC

**Figure 1:**
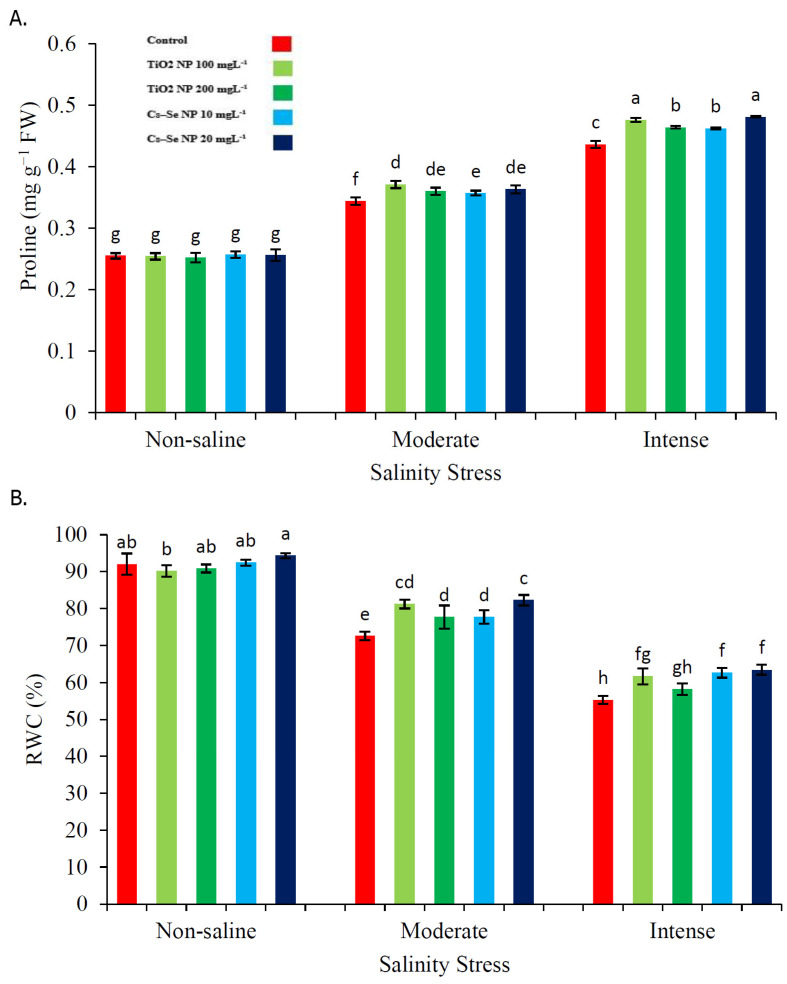
Effect of different concentrations of Cs–Se NPs and TiO_2_ NPs on the proline content (**A**) and RWC (**B**) of stevia (*Stevia rebaudiana* Bertoni) leaf under salinity stress. Data are the average of 3 repetitions ± standard error. Different letters indicate significantly different values according to Duncan’s post hoc analysis at *p* < 0.05.

Salinity significantly increased the proline content and decreased the relative water content (RWC) (Figure 1). The findings of the current study revealed that severe salinity increased the proline content by 41.51% and decreased the RWC by 39.94% in comparison with the non-saline condition, whilst treatments of TiO_2_ NPs and Cs–Se NPs resulted in higher levels of proline content relative to the 50 and 100 mM NaCl stress alone. Moreover, TiO_2_ NPs and Cs–Se NPs increased the RWC in plants under salinity stress. Our results showed that the treatment with Cs–Se NPs (20 mg L^−1^) significantly increased the proline content (5.23% and 9.35%) and RWC (11.73% and 12.83%) in comparison with non-treated plants under the 50 and 100 mM salt levels, respectively. Furthermore, TiO_2_ NPs (100 mg L^−1^) significantly increased the proline content (7.27% and 8.40%) and RWC (10.60% and 10.33%) as compared with the non-treated plants under 50 and 100 mM salt levels, respectively (Figure 1A,B).

### 2.4. Effect of Cs–Se NPs and TiO_2_ NPs on Leaf Content of MDA and H_2_O_2_, as Well as Electrolyte Leakage 

**Figure 2:**
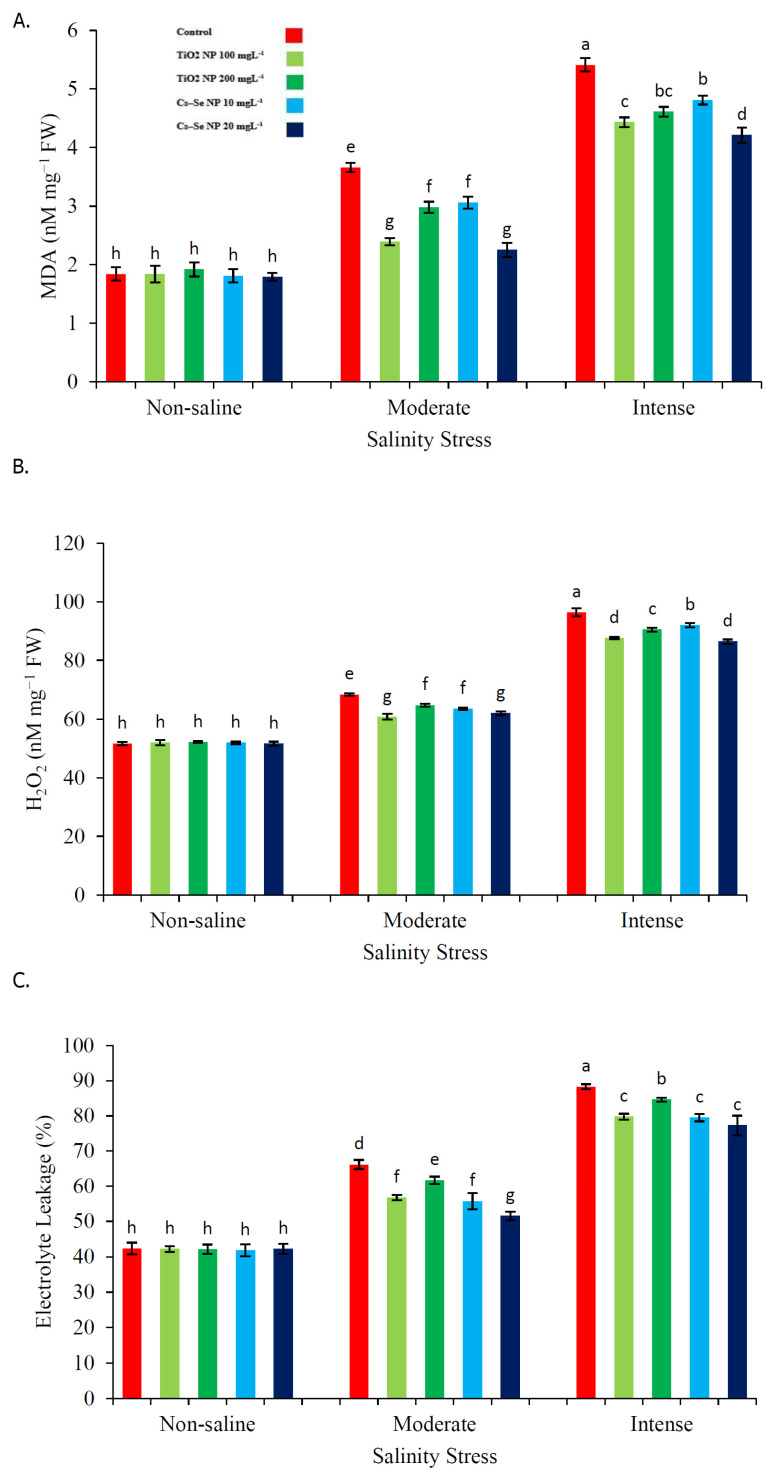
Effect of different concentrations of Cs–Se NPs and TiO_2_ NPs on the content of MDA (**A**), H_2_O_2_ (**B**) and the electrolyte leakage (%) parameter (**C**) of stevia (*Stevia rebaudiana* Bertoni) leaves under salinity stress. Data are the average of 3 repetitions ± standard error. Different letters indicate significantly different values according to Duncan’s post hoc analysis at *p* < 0.05.

As expected, salinity caused substantial increases in leaf MDA, H_2_O_2_ and electrolyte leakage. The highest leaf MDA (5.41 nM mg^−1^ FW), H_2_O_2_ content (96.40 nM mg^−1^ FW) and electrolyte leakage (88.30%) were observed under the severe salinity condition (Figure 2). For the case regarding the enhanced agronomic performance and photosynthetic pigment concentration, both treatments of Cs–Se NPs and TiO_2_ NPs substantially affected the leaf contents of MDA and H_2_O_2_, as well as electrolyte leakage. Corresponding to the treatments, Cs–Se NPs (20 mg L^−1^) significantly decreased the content of MDA (38.52% and 22.18%) and H_2_O_2_ (9.34% and 10.38%), as well as electrolyte leakage (22.06% and 12.45%), in relation to the non-treated plants grown under the 50 mM and 100 mM salinity levels, respectively. Moreover, the TiO_2_ NPs (100 mg L^−1^) treatment significantly decreased the content of MDA (34.69% and 18.11%) and H_2_O_2_ (11.16% and 9.11%), as well as electrolyte leakage (14.25% and 9.66%), as compared with the non-treated plants under 50 mM and 100 mM levels of salinity (Figure 2A–C).

### 2.5. Effect of Cs–Se NPs and TiO_2_ NPs on Total Phenolics Content and DPPH Scavenging Activity

**Figure 3:**
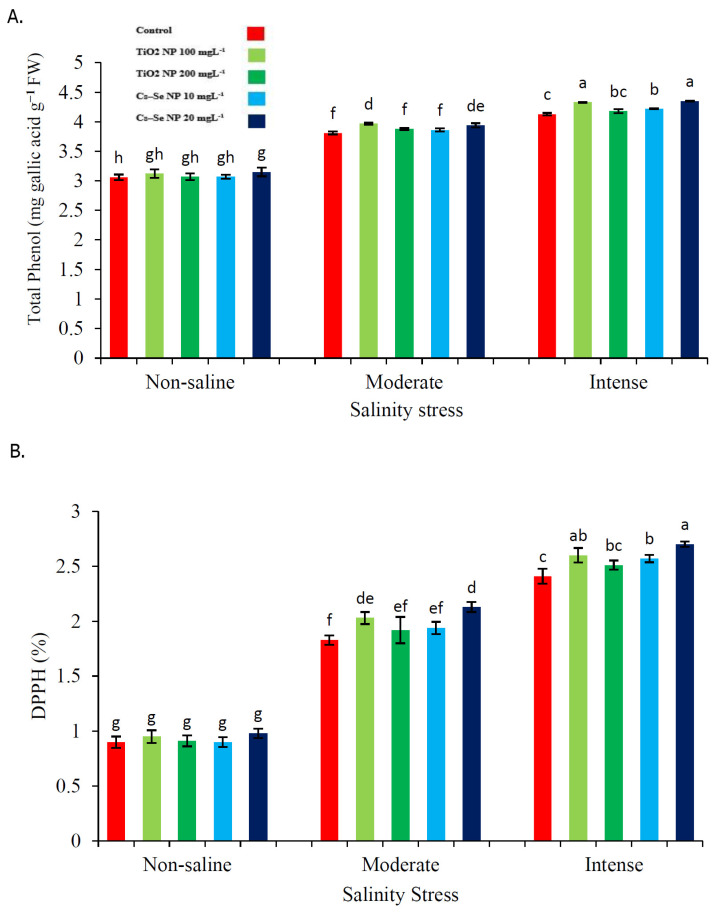
Effect of different concentrations of Cs–Se NPs and TiO_2_ NPs on the Total Phenol (**A**) and total antioxidant capacity (**B**) of stevia (*Stevia rebaudiana* Bertoni) leaves under salinity Stress. Data are the average of 3 repetitions ± standard error. Different letters indicate significantly different values according to Duncan’s post hoc analysis at *p* < 0.05.

The application of Cs–Se NPs (20 mg L^−1^) significantly increased the total phenolics content (3.24% and 5.07%) and total antioxidant capacity (14.08% and 10.74%) as compared with the non-treated plants under the 50 mM and 100 mM levels of salinity (Figure 3). Similar to the Cs–Se NPs (20 mg L^−1^), the TiO_2_ NPs (100 mg L^−1^) treatments also significantly increased the total phenolics content (3.97% and 4.63%) and DPPH radical scavenging activity (9.85% and 7.30%) as compared with the non-treated plants under the 50 mM and 100 mM levels of salinity (Figure 3A,B).

### 2.6. Effect of Cs–Se NPs and TiO_2_ NPs on Antioxidant Enzymes Activity

**Figure 4:**
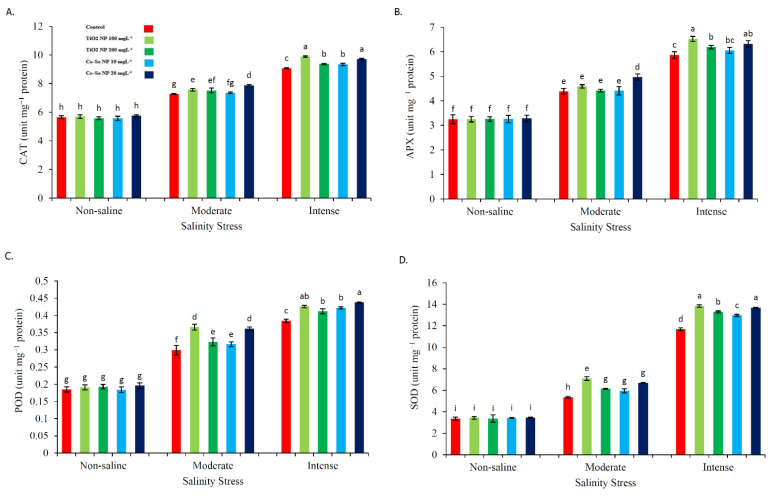
Effect of different concentrations of Cs–Se NPs and TiO_2_ NPs on the CAT (**A**), APX (**B**), POD (**C**), and SOD (**D**) enzyme activities of stevia (*Stevia rebaudiana* Bertoni) leaves under salinity stress. Data are the average of 3 repetitions ± standard error. Different letters indicate significantly different values according to Duncan’s post hoc analysis at *p* < 0.05.

Antioxidant enzymes protect cells from oxidative damage by eliminating excess reactive oxygen species in plants. Current findings showed that the application of Cs–Se NPs (20 mg L^−1^) significantly increased the CAT (7.61% and 6.49%), APX (11.67% and 7.12%), POD (17.17% and 12.32%) and SOD (20.11% and 14.69%) activity in comparison with non-treated plants under the 50 mM and 100 mM salinity levels. Moreover, TiO_2_ NPs (100 mg L^−1^) significantly improved the CAT (3.83% and 8.19%), POD (18.30% and 9.85%) and SOD (24.50% and 15.67%) activity as compared with the non-treated plants under the 50 and 100 mM NaCl conditions, respectively. In addition, TiO_2_ NPs (100 mg L^−1^) significantly increased APX (9.96%) activity as compared with the non-treated plants under 100 mM NaCl (Figure 4A–D).

### 2.7. Effect of Cs–Se NPs and TiO_2_ NPs on Essential Oil Content, as Well as Stevioside and Rebaudioside A Content

**Figure 5:**
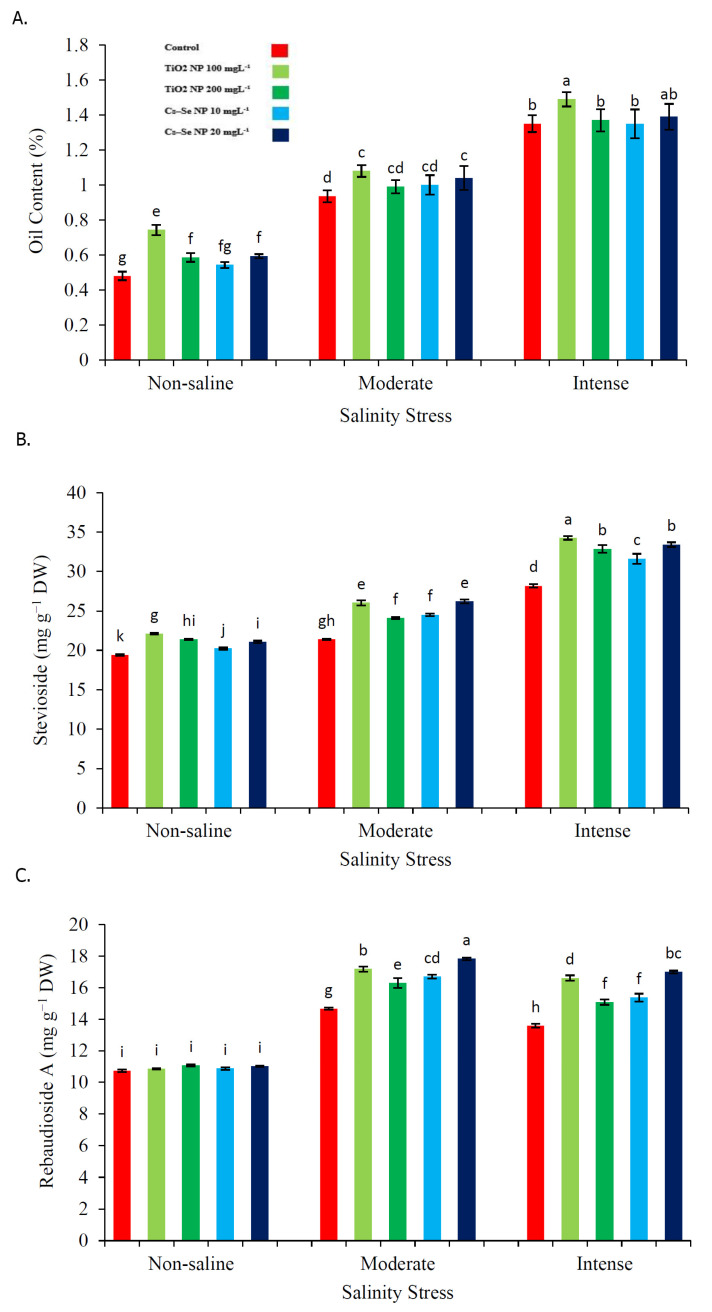
Effect of different concentrations of Cs–Se NPs and TiO_2_ NPs on the oil content (**A**), stevioside (**B**) and rebaudioside A (**C**) of stevia (*Stevia rebaudiana* Bertoni) leaves under salinity stress. Data are the average of 3 repetitions ± standard error. Different letters indicate significantly different values according to Duncan’s post hoc analysis at *p* < 0.05.

Salinity and NP treatments significantly affected the essential oil content, as well as the stevioside and rebaudioside A contents (Figure 5). Increasing the salinity stress level led to an increase in essential oil content. The interactive effects of the major treatments on essential oil content were also found to be significant. Specifically, the findings revealed that Cs–Se NPs (20 mg L^−1^) increased the essential oil level the most (10.00% and 2.87%) as compared with the non-treated plants under the 50 mM and 100 mM levels of salinity, respectively. Moreover, TiO_2_ NPs (100 mg L^−1^) significantly improved the essential oil content (13.33% and 9.39%) as compared with the non-treated plants under the 50 mM and 100 mM saline conditions (Figure 5A). Regarding stevioside and rebaudioside A, the stevioside content increased with the severity of the stress, whilst rebaudioside A content increased up to 50 mM NaCl and then showed a decrease. In addition to the salt-stress-induced increases in stevioside content, Cs–Se NPs (20 mg L^−1^) treatments also significantly increased the stevioside content (18.38% and 15.59%) and rebaudioside A content (17.76% and 20.05%) as compared with the non-treated plants under the 50 mM and 100 mM saline conditions, respectively. Similar to the treatment of Cs–Se NPs, the application of TiO_2_ NPs (100 mg L^−1^) significantly increased the stevioside content (17.81% and 17.66%) and rebaudioside A content (14.61% and 18.18%) as compared with the non-treated plants under the 50 mM and 100 mM saline conditions, respectively (Figure 5B,C).

### 2.8. Heat Map Clustering and Principal Component Analysis of the Examined Parameters

**Figure 6:**
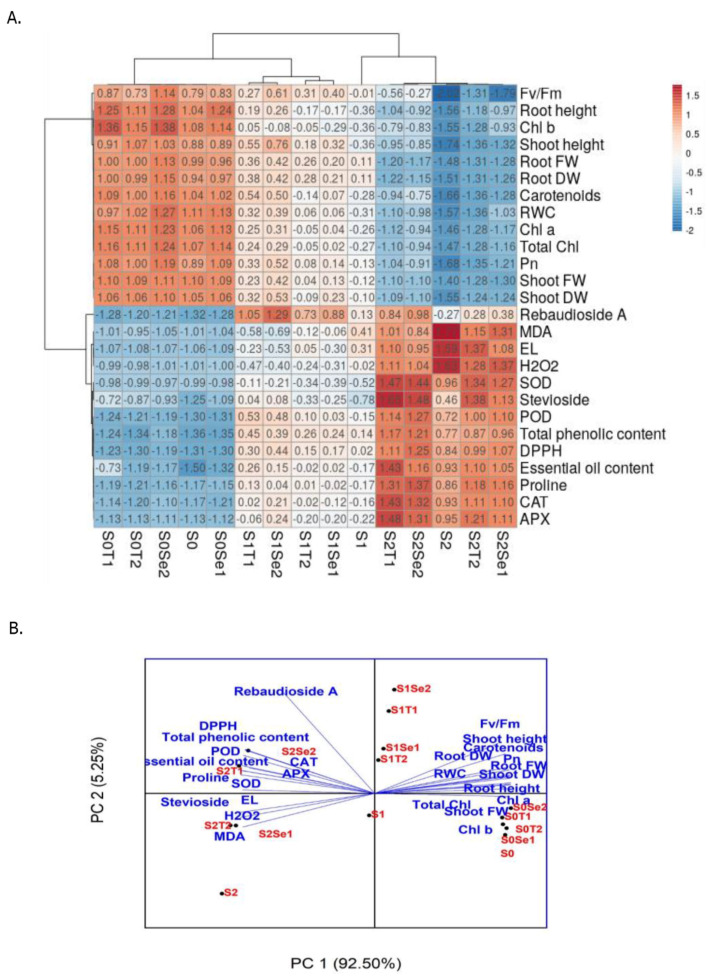
Heat map corresponding to the dependent and independent variables along with the treatments (**A**) and the principal component analysis regarding the observations (**B**) of salinity and TiO_2_ and Cs–Se nanoparticle applications on the examined traits in *Stevia rebaudiana* Bertoni (S_0_: 0 mM NaCl; S_0_Se_1_: 0 mM NaCl, 10 mg L^−1^ Cs–Se NPs; S_0_Se_2_: 0 mM NaCl, 20 mg L^−1^ Cs–Se NPs; S_0_T_1_: 0 mM NaCl, 100 mg L^−1^ TiO_2_ NPs; S_0_T_2_: 0 mM NaCl, 200 mg L^−1^ TiO_2_ NPs; S_1_: 50 mM NaCl; S_1_Se_1_: 50 mM NaCl, 10 mg L^−1^ Cs–Se NPs; S_1_Se_2_: 50 mM NaCl, 20 mg L^−1^ Cs–Se NPs; S_1_T_1_: 50 mM NaCl, 100 mg L^−1^ TiO_2_ NPs; S_1_T_2_: 50 mM NaCl, 200 mg L^−1^ TiO_2_ NPs; S_2_: 100 mM NaCl; S_2_Se_1_: 100 mM NaCl, 10 mg L^−1^ Cs–Se NPs; S_2_Se_2_: 100 mM NaCl, 20 mg L^−1^ Cs–Se NPs; S_2_T_1_: 100 mM NaCl, 100 mg L^−1^ TiO_2_ NPs; S_2_T_2_: 100 mM NaCl, 200 mg L^−1^ TiO_2_ NPs).

Due to the large amounts of data about the examined parameters corresponding to the salt stress and NPs treatments, a heat map was constructed in order to clarify, visualize and correlate the relevant findings of the current study (Figure 6A). Based on the analysis results, the clustering revealed that different combinations of salt stress (S_0_: control, S_1_: 50 mM NaCl and S_2_: 100 mM NaCl) and NPs treatments (T_1_: TiO_2_ NPs 100 mg L^−1^, T_2_: TiO_2_ NPs 200 mg L^−1^, Se_1_: Cs–Se NPs 10 mg L^−1^ and Se_2_: Cs–Se NPs 20 mg L^−1^) were classified into two major groups. As part of the clustering, all salt stress groups were clearly classified. The first group was composed of the higher level of salt stress (S_2_: 100 mM NaCl), whilst the second group was composed of the control (S_0_) and the lower level of salt stress (S_1_: 50 mM NaCl). Interestingly, the scattering of the NPs was similar to the salt stress groups corresponding to the responses of stevia plants. Considering the examined parameters of the plant, two major clusters were also observed. The heat map clustering was supported by principal component analysis (PCA), with a high explained variance ratio of the components (accounting for 97.75% of the variability of the original data) (Figure 6B). As deduced from both the heat map and the PCA, the examined parameters were clearly sorted into two groups. The first group was regarded as the yield parameters and photosynthesis-related parameters, whilst the second group included stress-related physiological and biochemical parameters, as well as secondary metabolites.

## 3. Discussion

In the present study, the agronomic and biochemical responses of stevia grown under salt stress and Cs–Se NPs and TiO_2_ NPs treatments were assessed. As previously reported for stevia, in particular [30,34,36], salinity stress reduces agronomic parameters, such as shoot and root length, shoot and root fresh weight and shoot and root dry weight, but the application of Cs–Se NPs (20 mg L^−1^) and TiO_2_ NPs (100 mg L^−1^) positively regulates the relevant parameters (shoot and root length, shoot and root fresh weight and shoot and root dry weight). Improvement in plant growth parameters might be explained by the NP-mediated enhancement in the performances of photosynthesis traits, such as chlorophyll *a* and *b*, total chlorophyll, carotenoid content, Pn and Fv/Fm under salinity conditions, as deduced from the present findings. Regarding TiO_2_ NPs, the affirmative acts of the relevant NPs were also noted for Moldavian balm [25] and broad bean [28], which were correlated with the contribution to the chlorophyll development and Rubisco activities [37]. As formerly reported by Yang et al. [37] and Frazier et al. [38], the upregulation of gene expression and activity of Rubisco enzyme was provided by TiO_2_ NPs treatments. Moreover, Tumburu et al. [39] reported that TiO_2_ NPs increased the expression of genes related to photosynthetic metabolism in leaves of the *Arabidopsis thaliana* plant. TiO_2_ NPs also increased photosynthesis parameters by increasing the light energy of the PSI absorbed by the chloroplast membrane to be transferred to PSII, the promotion of light energy conversion to electron energy and the electron transport and acceleration of water photolysis and oxygen evolution [40]. In addition to activation of the photosynthesis machinery of the plant, growth parameters are also positively correlated with the absorption of essential elements in the Cs–Se-NP- and TiO_2_-NP-treated plants under salinity conditions [27,41]. 

Considering Se NP applications, the current findings are consistent with the reports indicating that Se NPs significantly improved the growth and photosynthetic performances of strawberry [42] and tomato [43] plants under salinity stress conditions. As deduced from the former reports, TiO_2_ NPs and Cs–Se NPs have a positive regulatory role on the photosynthetic system and growth parameters.

Munns and Tester [44] reported that reduced water uptake due to increased osmolarity of soil solution is one of the earliest effects of salinity on plants. However, the water balance of the plants can be maintained by increasing their osmolyte (e.g., proline) levels. In addition to the maintained water status of the cells, Shamsul et al. [45] reported that the overaccumulation of proline might provide several benefits concerned with scavenging ROS to prevent a sustained oxidative burst and stabilizing membranes to prevent electrolyte leakage. Along with the treatments of Cs–Se NPs (20 mg L^−1^) and TiO_2_ NPs (100 mg L^−1^), notable increases were recorded for the proline content and RWC in stevia. As is well-known, salinity causes substantial decreases in the assimilation, accumulation and metabolism of nitrogen, which has a key role in the biosynthesis of proline [46]. However, Se NPs and TiO_2_ NPs treatments might elevate the proline content in plants through increasing the activity of nitrate reductase (a key enzyme of nitrogen assimilation) [29,37,47] and plant nitrogen status [48,49]. As in the case of enhanced growth parameters, current findings are also consistent with the reports indicating that the application of Cs–Se NPs [27], Se NPs [42] and TiO_2_ NPs [28] increased proline and RWC in bitter melon, strawberry and broad bean, respectively, under salinity stress conditions. 

As is well reported for quite a number of plants, salinity stress causes excessive ROS production, which then attacks lipids, proteins, DNA and carbohydrates. Furthermore, an oxidative burst results in membrane lipid peroxidation (oxidative damages) and the production of MDA in plants [50]. In order to maintain their proper and sustainable development, plants have evolved two detoxification mechanisms, namely, enzymatic and non-enzymatic antioxidants defense mechanisms, in order to maintain ROS at safe levels. In this regard, CAT, SOD, POD and APX are some of the principal enzymatic antioxidants in plants [51], whilst phenolic compounds are one of the main non-enzymatic defense compounds [52]. As is well-documented in quite a number of studies, the total antioxidant capacity of plants is correlated with the phenolic contents available [53,54,55,56]. As in the current study, we observed that the application of Cs–Se NPs (20 mg L^−1^) and TiO_2_ NPs (100 mg L^−1^) significantly increased the total phenolics content; CAT, SOD, POD and APX activity; and total antioxidant capacity. The application of Se NPs (20 mg L^−1^) and TiO_2_ NPs (100 mg L^−1^) also significantly decreased H_2_O_2_, MDA content and EL in the stevia plants under salinity conditions. As previously reported for bitter melon under salinity stress, Cs–Se NPs increased the total phenols; CAT, SOD, POD and APX activity; and total antioxidant capacity; and decreased the H_2_O_2_ and MDA content, as well as the EL [27]. Zahedi et al. [42] reported that Se NPs improved POD and SOD activity and decreased H_2_O_2_ and MDA levels in strawberries under salinity conditions. Moreover, Gohari et al. [25] observed that the application of TiO_2_ NPs increased the CAT, SOD, GP (guaiacol peroxidase) and APX activity and decreased H_2_O_2_ content in Moldavian balm plant under salinity conditions. In addition, Abdel Latef et al. [28] reported that the application of TiO_2_ NPs increased SOD and APX activity and decreased MDA content in broad bean plants under saline conditions. Considering the relevant findings of detoxification elements examined as part of this study, the increases in activity of the enzymes and phenolic contents might explain the improved physiology and agronomic traits of stevia.

Considering medicinal plants, the pharmaceutical activities or biological activities of these relevant plant species are dependent on the metabolites available [57,58,59]. In this regard, to increase or to keep the desired level of the medicinally important metabolites is one of the great interests of stevia researchers [30,32,60]. Out of the medicinal plants defined, stevia is one of the reputed medicinal plants used in the pharmaceutical industry, with it being well-known for its diabetes-controlling properties. Stevioside and rebaudioside A are the most important compounds of stevia. Steviol glycoside is a group of secondary metabolites that are derived from the mono-, di- and tetra-terpene biosynthetic pathways. The major steviol glycosides, such as stevioside and rebaudioside A, are non-caloric sweeteners that are used in many countries due to being sweeter than sucrose [61]. In this context, any exogenous treatments for stimulating the production of these metabolites might be of great interest to stevia growers. Along with the present study, we observed an increase in stevioside content but a decrease in rebaudioside A content under severe salt stress. Of the available reports with respect to the impacts of salt stress on stevioside and rebaudioside A content, Zeng et al. [30] showed that salt stress reduced the content of stevioside and rebaudioside A, whilst Cantabella et al. [32] reported that salinity increased rebaudioside A content in stevia plants. Moreover, osmoprotectant functions of those metabolites were reported under salt stress [32] and water stress [61]. In this study, we also recorded a substantial increase in stevioside content under severe saline conditions, which are consistent with the previous reports [32,61]. Regarding this study, the application of Cs–Se NPs (20 mg L^−1^) and TiO_2_ NPs (100 mg L^−1^) significantly increased the stevioside and rebaudioside A contents. In addition to these metabolites, the relevant NP treatments also augmented the essential oil content of stevia. In the report by Sheikhalipour et al. [27], Cs–Se NPs increased the essential oil content in bitter melon fruits under salinity conditions. Moreover, TiO_2_ NPs increased the essential oil content in *Salvia* *officinalis* [62], *Mentha piperita* [63] and Moldavian balm [25]. In addition, the application of Se NPs increased the synthesis of secondary metabolites through increases in the expression of biosynthesis pathway-related genes: Pal, 4CL, HCT, pAmt, Kas, Acl, Fat and AT3 in pepper [64], and Pal and 4CL in bitter melon plant [65]. The modulator roles of NPs on the pathways of plant secondary metabolites are not well-explained hitherto even though in this regard, Marslin et al. [66] postulated that the penetration and/or fixation of NPs on the cell surface and/or within cells causes elevated levels of ROS. Subsequently, cytoplasmic Ca^2+^, antioxidant system and mitogen-activated protein kinase (MAPK) cascades trigger transcriptional reprogramming of relevant genes involved in secondary metabolism.

## 4. Materials and Methods

### 4.1. Preparation of Titanium Dioxide Nanoparticles and Selenium Functionalized Using Chitosan Nanoparticles

The synthesis and characterization of the selenium functionalized using chitosan nanoparticles (Cs–Se NPs) and titanium dioxide nanoparticles (TiO_2_ NPs) used in this experiment are described in Sheikhalipour et al. [27] and Gohari et al. [25]. The synthesis was carried out in a nanochemistry laboratory, University of Maragheh, Iran. Regarding the characterization results, the sizes of the Cs–Se and TiO_2_ NPs were 60 nm and 25 nm, respectively.

### 4.2. Plant Material and Treatments

The experiment was conducted in the research greenhouses of the Faculty of Agriculture, Mohaghegh Ardabili University (46°16′ E, 37°23′ N, altitude 1485 m), as a factorial experiment using a random design. As a plant material, vegetatively propagated *Stevia rebaudiana* Bertoni cuttings with three fully-developed leaves were obtained from Pakanbazr Company, Isfahan, Iran. The seedlings were transferred to main pots (40 cm × 15 cm) containing coco peat and perlite (2:1, *v*/*v*) and uniformly irrigated with tap water each day for one week, then fertigated with half-strength Hoagland’s nutrient solution every 2 days until harvest. Thereafter, the plants were continuously watered with full-strength Hoagland’s nutrient solution supplemented with NaCl at concentrations of 0, 50 and 100 mM (non-saline conditions, moderate and intense stress) two weeks after being transferred to the main pots, which continued up to plant harvest (prolonged stress of approximately forty days after applying salt stress). To prevent the accumulation of salt in the culture medium, the culture medium was washed once a week with tap water. After two weeks from the beginning of salinity stress, the plants were sprayed with selenium nanoparticles (Cs–Se NPs) at concentrations of 0, 10 and 20 mg L^−1^ and anatase titanium dioxide nanoparticles (TiO_2_ NPs) at concentrations of 0, 100 and 200 mg L^−1^. Selenium nanoparticles (Cs–Se NPs) and titanium dioxide nanoparticles (TiO_2_ NPs) were applied once a week during the growth period (three times). All treatments were dispersed in deionized water (DIW) and then Tween 20 (Sigma-Aldrich Co, St. Louis, MO, USA) was added to the suspension and foliar application was performed. This experiment was performed in three repetitions and there were three plants in each repetition (9 plants for each treatment). The greenhouse temperatures of 26/19 ± 4 ^°^C (day/night) and air relative humidity of ca. 80 ± 5% were maintained throughout the experiment under natural light and length of the day.

### 4.3. Plant Growth and Relative Water Content (RWC) in Leaves

Shoot and root height were recorded at the harvest stage. The fresh weight (FW) of the shoots and roots was recorded at harvest and the shoot and root dry weight (DW) was measured after samples were oven-dried (UFP800, Memmert, Büchenbach, Germany) at 70 °C for 72 h. The RWCs of leaves in treated and non-treated plants were determined using the method of Sairam and Srivastava [67].

### 4.4. Photosynthetic Pigments, Gas Exchange Capacity and Chlorophyll Fluorescence

Chlorophyll *a* (Chl *a*) and chlorophyll *b* (Chl *b*), total chlorophyll and carotenoids (Car) were extracted from fresh leaves (0.2 g) using 80% (*v*/*v*) acetone. After centrifugation (15,000× *g* for 5 min at 25 °C), the absorbance for each extract was spectrophotometrically recorded at 470, 646 and 663 nm (UV-1800 Shimadzu, Kyoto, Japan), and the concentration of photosynthetic pigments was determined using the following equations from Arnon [68]:

Chl *a* = (12.47 × A663) − (3.62 × A645) (1)

Chl *b* = (25.06 × A645) − (6.5 × A663) (2)

Carotenoids = (1000 × A470) − (1.29 Chl *a* − 53.78 Chl *b*) (3)

Photosynthetic rates (Pn) of leaves at the second or third nodes of the plants were measured using an infrared (IR) gas analyzer (LI-6400T, Li-Cor Inc., Lincoln, NE, USA), with a red/blue light source (6400-02B) [69]. The chlorophyll fluorescence parameter (Fv/Fm) was measured using a DUAL-PAM-100 chlorophyll fluorometer (Heinz Walz, Efeltrich, Germany) after the adaption of stevia in the dark for 30 min. Chlorophyll fluorescence was determined on sunny days between 8:00 h and 9:00 h [70].

### 4.5. Proline, Malondialdehyde (MDA) and Hydrogen Peroxide (H_2_O_2_) Contents and Electrolyte Leakage (EL)

The leaf proline content was quantified according to the method of Bates et al. [71]. Briefly, fresh leaves were homogenized in a 3% sulfosalicylic acid solution. Then, the homogenates were centrifuged at 15,000× *g* for 10 min at 4 °C. After centrifugation, 1 mL of the supernatant was placed in a tube and was allowed to react with 1 mL acid ninhydrin and 1 mL of glacial acetic acid. The relevant mixture was heated at 100 °C for 60 min. The reaction was terminated by placing the mixture on ice, then 2 mL of toluene was used for extracting the reaction mixture. The separation of the two phases was carried out after keeping the samples at room temperature for 30 min. Finally, the absorbances of the upper phase were read using a spectrophotometer at 520 nm and toluene was used as the blank. 

Lipid peroxidation was measured using the amount of malondialdehyde (MDA) [72]. The samples of fresh leaf tissue (0.3 g) were ground in 20% trichloroacetic acid and centrifuged at 13,000 rpm for 15 min and 4 mL of 20% TCA were added to 1 mL of the supernatant. The mixtures were heated for 30 min in a hot water bath (95 °C) and were thereafter immediately cooled in an ice bath. Malondialdehyde content was determined at two wavelengths of 532 and 600 nm. To calculate the MDA concentration, a molar absorption coefficient of 155 mM^−1^ cm^−1^ was used. 

For the quantification of hydrogen peroxide (H_2_O_2_), the method proposed by Alexieva et al. [73] was employed, where hydrogen peroxide was measured spectrophotometrically after reacting with KI. The reaction mixture consisted of 0.5 mL 0.1% trichloroacetic acid (TCA) leaf extract supernatant, 0.5 mL of 100 mM K-phosphate buffer and 2 mL reagent (1 MKI *w*/*v* in fresh double-distilled water). The blank probe consisted of 0.1% TCA in the absence of leaf extract. The reaction was developed for 1 h in darkness and the absorbance was measured at 390 nm. The amount of hydrogen peroxide was calculated using a standard curve prepared with known concentrations of H_2_O_2_. 

Electrolyte leakage, as an indicator of stress damage, was determined according to Nanjo et al. [74]. Samples were kept in falcons comprising 10 mL of distilled water at room temperature (25 °C) and shook for 24 h at 120 rpm. The primary electrical conductivity of solution (EC_1_) was recorded. The electrolytes of the tissue were released by autoclaving the same samples at 100 °C for 2 h. Then, the solution was cooled at room temperature and the electrical conductivity of the solution (EC_2_) was registered. The relative electrolyte leakage EC_1_/EC_2_ × 100 was recorded. 

### 4.6. Antioxidant Enzymes

Fresh leaf samples (0.5 g) were homogenized in 5 mL of 0.05 M phosphate buffer (1 mM EDTA, 1% PVP, pH 7.8) and the homogenates were centrifuged at 12,000× *g* for 20 min at 4 °C. The supernatants were collected and used for the determination of peroxidase (POD), ascorbate peroxidase (APX), catalase (CAT) and superoxide dismutase (SOD) activity. The activity of POD was measured using the method of Hemeda and Klein [75]. To determine the peroxidase activity, a reaction mixture containing enzyme extract, 100 mM potassium phosphate buffer (pH 6.0), 5 µL of 10% (*w*/*v*) H_2_O_2_ and 16 mM guaiacol was used. The enzyme activity was expressed at 470 nm for 1 min as millimoles of produced tetraguaiacol per minute per milligram of soluble protein (U mg^−1^). The activity of CAT was measured using the method of Aebi [76]. The reaction mixture contained 25 mM phosphate buffer (pH 6.8) and 10 mM H_2_O_2_. The reduction at 240 nm was registered. The activity of SOD was measured using the method of Giannopolitis and Ries [77]. One unit of SOD activity was defined as the amount of enzyme required to cause 50% inhibition of the reduction rate of NBT (nitro blue tetrazolium) at 560 nm. The activity of APX was measured using the method of Nakano and Asada [78]. To determine the ascorbate peroxidase activity, the reaction mixture contained enzyme extract, 50 mM phosphate buffer (pH 7.0), 0.5 mM AsA, 1 mM H_2_O_2_ and 0.1 mM EDTA. The reaction was started via the addition of H_2_O_2_ and the reduction in absorbance at 290 nm was recorded for 1 min.

### 4.7. Extraction and Quantification of Total Phenolics Content and Radical Scavenging Activity (DPPH)

The air-dried and powdered samples (0.5 g) were extracted using 3 mL 85% (*w/w*) methanol and then centrifuged at 12,000× *g* for 15 min. This methanolic extract was used to measure the total phenolic content and radical scavenging activity (DPPH). The total phenolic concentration was assayed used the Folin–Ciocalteu reagent, as described by Chun et al. [79]. For the total antioxidant capacity assay, the DPPH scavenging activities of the extracts were determined according to the method proposed by Suja et al. [80], which involved preparing a 0.1 mM solution of 2.2-diphenyl-1-picrylhydrazyl (DPPH) in absolute ethanol. The antioxidant activity (%) was determined using the following formula: A_0_ − A_1_/A_0_ × 100, where A_0_—the absorbance of the control and A_1_—the absorbance of the standard.

### 4.8. Stevioside, Rebaudioside A and Essential Oil Contents

The contents of essential oils (mL 100 g^−1^ FW) were measured in a Clevenger-type apparatus. A total of 0.1 g of powdered dried leaves were transferred to 15 mL tubes, 3 mL distilled water was added and the mixture was kept in a water bath for 30 min at 80 °C. The resultant solution was firstly centrifuged at 12,000× *g* for 5 min and the supernatant was recovered (this process was repeated three times). The volume of the final supernatant was diluted to exactly 10 mL using distilled water and filtered using a 0.45 m nylon filter attached to a syringe. Then, the quantifications of the stevioside and rebaudioside A were performed using HPLC (Unicam Crystal 200, Thermo Fisher, UK) with a diode array detector based on the method of Martins et al. [81]. The main part of the mobile phase consisted of acetonitrile (80% *w*/*w*) buffered to pH = 0.5 with 100 mL of 0.02 M glacial acetic acid and 200 mL of 0.1 M sodium hydroxide for every 500 mL of the total solvent. The injection volume of 5 mL and a constant flow rate of 0.7 mL/min were programmed to flow through the Agilent Zorbax column (250 mm × 9 mm × 4.6 mm, 51 min) in gradient mode varying from 10:90 to 90:10 *v*/*v* following the detection using UV at 210 nm.

### 4.9. Statistical Analysis

Data were analyzed by using SPSS 20.0 software and all data were statistically analyzed using Duncan’s multi-range test with *p* < 0.05 as the significant difference level. Moreover, a principal component analysis (PCA) was performed in order to discriminate the treatments of nanoparticles and salinity on the basis of agronomic traits, physiological attributes, enzymatic activities and the contents of stevioside and rebaudioside A (OriginLab Software). Furthermore, a heat map corresponding to the findings from the treatments was constructed for visualizing and relating the dependent and independent variables (ClustVis).

## 5. Conclusions

Nanotechnology is a new method for increasing plant tolerance against biotic and abiotic stresses. The results of this experiment showed that the application of TiO_2_ NPs and Cs–Se NPs increased the growth parameters by increasing photosynthetic parameters under salinity stress conditions. Moreover, the application of TiO_2_ NPs and Cs–Se NPs increased the proline content, which led to an increase in the RWC in the plants. Furthermore, the application of TiO_2_ NPs and Cs–Se NPs reduced the oxidative damage in the plants through increased enzymatic activity and non-enzymatic antioxidants compound content in stevia under salt stress conditions. Finally, the application of TiO_2_ NPs and Cs–Se NPs increased the essential oil content and concentration of stevioside (in non-saline and saline conditions) and rebaudioside A (under saline conditions) in stevia plants. Generally, the application of TiO_2_ NPs (100 mg L^−1^) and Cs–Se NPs (20 mg L^−1^) increased the measured traits of stevia more than TiO_2_ NPs (200 mg L^−1^) and Cs–Se NPs (10 mg L^−1^). Therefore, the use of TiO_2_ NPs (100 mg L^−1^) and Cs–Se NPs (20 mg L^−1^) might be regarded as a useful strategy for increasing growth, antioxidant activity, essential oil content and steviol glycosides concentration in stevia under saline conditions.

## Figures and Tables

**Table 1:** Effects of foliar application of Cs–Se NPs (0, 10 and 20 mg L^−1^) and TiO_2_ NPs (0, 100 and 200 mg L^−1^) on some growth and photosynthesis parameters of stevia (*Stevia rebaudiana* Bertoni) under different salt stress (0, 50 and 100 mM NaCl) treatments.

NaCl (mM)	Treatments	Shoot Height (cm)	Root Height cm)	Shoot FW (g)	Shoot DW (g)	Root FW (g)	Root DW (g)	Chl *a* (mg g^−1^ FW)	Chl *b* (mg g^−1^ FW)	Total Chl (mg g^−1^ FW)	Carotenoids (mg g^−1^ FW)	Fv/Fm	Pn (μmol m^−2^ s^−1^)
	No Treatment	46.72 ± 1.74 ^ab^	17.06 ± 0.40 ^a^	107.99 ± 4.50 ^a^	20.09 ± 0.67 ^a^	30.08 ± 0.17 ^a^	5.86 ± 0.04 ^a^	6.39 ± 0.18 ^a^	0.814 ± 0.012 ^a^	7.21 ± 0.18 ^a^	5.91 ± 0.12 ^a^	0.775 ± 0.010 ^b^	9.23 ± 0.04 ^c^
	TiO_2_ NPs 100 mg L^−1^	46.93 ± 2.64 ^ab^	17.56 ± 0.21 ^a^	107.88 ± 6.12 ^a^	20.11 ± 0.94 ^a^	30.15 ± 0.23 ^a^	5.93 ± 0.03 ^a^	6.50 ± 0.21 ^a^	0.835 ± 0.011 ^a^	7.33 ± 0.22 ^a^	5.93 ± 0.05 ^a^	0.778 ± 0.006 ^b^	9.38 ± 0.01 ^ab^
0	TiO_2_ NPs200 mg L^−1^	48.11 ± 0.74 ^a^	17.23 ± 0.45 ^a^	107.75 ± 6.74 ^a^	20.12 ± 1.11 ^a^	30.16 ± 0.30 ^a^	5.92 ± 0.06 ^a^	6.44 ± 0.18 ^a^	0.819 ± 0.006 ^a^	7.26 ± 0.18 ^a^	5.89 ± 0.13 ^a^	0.772 ± 0.007 ^b^	9.32 ± 0.01 ^bc^
	Cs–Se NPs 10 mg L^−1^	46.80 ± 1.72 ^ab^	17.55 ± 0.32 ^a^	107.69 ± 3.69 ^a^	20.15 ± 0.91 ^a^	29.92 ± 1.57 ^a^	5.89 ± 0.28 ^a^	6.48 ± 0.29 ^a^	0.819 ± 0.003 ^a^	7.29 ± 0.29 ^a^	5.90 ± 0.09 ^a^	0.776 ± 0.003 ^b^	9.39 ± 0.06 ^ab^
	Cs–Se NPs 20 mg L^−1^	47.81 ± 1.49 ^a^	17.65 ± 0.23 ^a^	108.37 ± 1.82 ^a^	20.30 ± 0.45 ^a^	30.97 ± 0.41 ^a^	6.12 ± 0.10 ^a^	6.59 ± 0.14 ^a^	0.836 ± 0.012 ^a^	7.43 ± 0.13 ^a^	5.97 ± 0.09 ^a^	0.789 ± 0.004 ^a^	9.48 ± 0.02 ^a^
	No Treatment	37.67 ± 1.57 ^e^	13.67 ± 0.23 ^c^	74.87 ± 3.44 ^d^	14.38 ± 0.41 ^c^	24.60 ± 0.41 ^c^	4.86 ± 0.08 ^c^	4.83 ± 0.09 ^e^	0.710 ± 0.008 ^d^	5.54 ± 0.08 ^d^	5.27 ± 0.05 ^d^	0.741 ± 0.000 ^e^	8.41 ± 0.03 ^g^
	TiO_2_ NPs 100 mg L^−1^	44.29 ± 0.84 ^bcd^	15.01 ± 0.15 ^b^	84.31 ± 2.49 ^bc^	16.48 ± 0.26 ^b^	26.15 ± 0.21 ^bc^	5.18 ± 0.03 ^bc^	5.43 ± 0.08 ^bc^	0.739 ± 0.008 ^b^	6.17 ± 0.09 ^b^	5.67 ± 0.07 ^b^	0.752 ± 0.001 ^d^	8.78 ± 0.03 ^e^
50	TiO_2_ NPs 200 mg L^−1^	41.60 ± 1.94 ^d^	14.13 ± 0.25 ^c^	79.11 ± 0.77 ^cd^	14.43 ± 1.00 ^c^	25.49 ± 1.42 ^bc^	5.06 ± 0.29 ^bc^	5.08 ± 0.11 ^de^	0.732 ± 0.010 ^bc^	5.81 ± 0.12 ^c^	5.34 ± 0.05 ^cd^	0.754 ± 0.002 ^d^	8.58 ± 0.06 ^f^
	Cs–Se NPs 10 mg L^−1^	42.66 ± 1.56 ^cd^	14.12 ± 0.48 ^c^	81.60 ± 1.83 ^cd^	16.04 ± 0.50 ^bc^	25.16 ± 1.06 ^bc^	4.97 ± 0.24 ^bc^	5.19 ± 0.11 ^cd^	0.715 ± 0.004 ^cd^	5.90 ± 0.11 ^c^	5.44 ± 0.08 ^c^	0.758 ± 0.000 ^cd^	8.62 ± 0.03 ^f^
	Cs–Se NPs 20 mg L^−1^	45.83 ± 1.17 ^abc^	15.16 ± 0.10 ^b^	89.41 ± 1.66 ^b^	17.51 ± 0.16 ^b^	26.50 ± 0.73 ^b^	5.23 ± 0.14 ^b^	5.50 ± 0.19 ^b^	0.730 ± 0.001 ^bcd^	6.23 ± 0.19 ^b^	5.65 ± 0.11 ^b^	0.767 ± 0.004 ^bc^	8.93 ± 0.04 ^d^
	No Treatment	27.64 ± 1.70 ^h^	10.67 ± 0.19 ^f^	40.31 ± 0.86 ^f^	7.21 ± 0.28 ^e^	14.66 ± 0.48 ^e^	2.89 ± 0.04 ^e^	3.41 ± 0.08 ^h^	0.623 ± 0.006 ^f^	4.04 ± 0.07 ^g^	4.61 ± 0.02 ^g^	0.655 ± 0.004 ^i^	7.16 ± 0.04 ^l^
	TiO_2_ NPs 100 mg L^−1^	33.41 ± 0.14 ^fg^	12.03 ± 0.14 ^de^	48.42 ± 1.10 ^e^	9.48 ± 0.40 ^d^	16.41 ± 0.69 ^d^	3.25 ± 0.14 ^d^	3.82 ± 0.02 ^fg^	0.678 ± 0.001 ^e^	4.50 ± 0.02 ^ef^	4.96 ± 0.03 ^e^	0.717 ± 0.005 ^g^	7.67 ± 0.04 ^i^
100	TiO_2_ NPs200 mg L^−1^	30.45 ± 0.88 ^gh^	11.68 ± 0.05 ^e^	43.50 ± 0.57 ^ef^	8.76 ± 0.29 ^de^	15.75 ± 0.50 ^e^	3.13 ± 0.11 ^de^	3.62 ± 0.01 ^gh^	0.643 ± 0.014 ^f^	4.27 ± 0.02 ^fg^	4.76 ± 0.03 ^fg^	0.686 ± 0.005 ^h^	7.42 ± 0.03 ^k^
	Cs–Se NPs 10 mg L^−1^	30.68 ± 0.60 ^gh^	12.19 ± 0.09 ^de^	42.80 ± 0.42 ^ef^	8.73 ± 0.52 ^de^	15.92 ± 0.20 ^de^	3.19 ± 0.04 ^de^	3.75 ± 0.03 ^g^	0.668 ± 0.013 ^e^	4.42 ± 0.04 ^f^	4.79 ± 0.03 ^f^	0.665 ± 0.004 ^i^	7.53 ± 0.05 ^j^
	Cs–Se NPs 20 mg L^−1^	34.10 ± 0.14 ^f^	12.31 ± 0.08 ^d^	48.24 ± 0.86 ^e^	9.45 ± 0.74 ^d^	16.57 ± 0.61 ^d^	3.34 ± 0.10 ^d^	4.03 ± 0.05 ^f^	0.675 ± 0.013 ^e^	4.70 ± 0.04 ^e^	5.05 ± 0.03 ^e^	0.730 ± 0.006 ^f^	7.78 ± 0.05 ^h^

Data are the average of 3 repetitions ± standard error. Different letters in a column indicate significantly different values according to Duncan’s post hoc analysis at *p* < 0.05.

## Data Availability

The data that support the findings of this study are available from the corresponding author upon reasonable request.

## Sample Availability

Samples of the compounds are available from the authors.
